# Perception of and anxiety about COVID-19 infection and risk behaviors for spreading infection: an international comparison

**DOI:** 10.1186/s12991-021-00334-6

**Published:** 2021-02-18

**Authors:** Akihiro Shiina, Tomihisa Niitsu, Osamu Kobori, Keita Idemoto, Tasuku Hashimoto, Tsuyoshi Sasaki, Yoshito Igarashi, Eiji Shimizu, Michiko Nakazato, Kenji Hashimoto, Masaomi Iyo

**Affiliations:** 1 Center for Forensic Mental Health, Chiba University, 260-8670 Inohana 1-8-1, Chuo-ku, Chiba, Japan; 2 Department of Psychiatry, Graduate School of Medicine, Chiba University, Chiba, Japan; 3 Department of Psychology, International University of Health and Welfare, Akasaka, Japan; 4 Department of Psychiatry, School of Medicine, International University of Health and Welfare, Narita, Japan; 5 Department of Cognitive Behavioral Physiology, Graduate School of Medicine, Chiba University, Chiba, Japan

**Keywords:** COVID-19, Anxiety, Precautionary behaviors, International comparison, Questionnaire survey

## Abstract

**Background:**

To control the spread of the new SARS-CoV-2 infection's disease (COVID-19), appropriate precautionary behaviors by the public should be promoted. There are international differences in public cognitive and behavioral pattern, attitudes toward information sources, and anxiety about COVID-19. Information about these differences could increase understanding of the patterns of epidemic-related anxiety and behavior, and would help optimize future policies for preventing the next wave of the epidemic.

**Methods:**

To examine between-country differences in perception, attitude, and precautionary behaviors toward COVID-19, we conducted a cross-sectional study using an online questionnaire survey. Participants were adults who had been registered in Cross Marketing Group Inc. and living in the UK, Spain, or Japan. A total of 8,000 people stratified by age were recruited on a first-come, first-serve basis. Knowledge of and anxiety about COVID-19, the frequency of access and perceived credibility of several information sources, and the frequency of each precautionary behavior were examined on March 27–28, 2020, in Japan and April 17–21, 2020, in the UK and Spain.

**Results:**

Knowledge, anxiety, and the frequency of precautionary behaviors were higher in the UK and Spain than in Japan. Participants with infected acquaintances were more concerned about COVID-19. However, participants in the UK rarely wore a medical mask. Participants in the UK and Spain were more eager to obtain information about COVID-19 than those in Japan. Participants in Spain tended not to trust official information and to believe specialists’ comments instead.

**Conclusion:**

The rapidity of the spread of COVID-19, cultural background, and recent political situations seemed to contribute to the international differences here.

## Background

In December 2019, a novel species of coronavirus that causes very specific and critical pneumonia was identified: SARS-CoV-2. The virus spread worldwide within several months, leading to millions of infections, and the World Health Organization (WHO) declared a pandemic state on March 11, 2020. Citizens in many countries now face the risk of serious disease caused by SARS-CoV-2, i.e., COVID-19. Precautionary behaviors are important in reducing the risk of COVID-19 transmission. However, there are differences between countries in public attitudes, behaviors, and anxiety toward COVID-19.

Facing uncertain situations can increase anxiety levels, especially if there is potential mortality risk. Anxiety may lead both healthy and vulnerable individuals to engage in behaviors to protect themselves from contracting the virus [1]. Jones and Salathé reported that engagement in protective behaviors shows individual variation and may be affected by several factors [2]. A community-based survey by Harper et al. indicated that fear of COVID-19 was the only predictor of positive behavior changes [3].

Recent studies suggest that some super-spreaders unintentionally transmit the virus because of their hyper-activity [4, 5]. These individuals are unlikely to be anxious about infection and tend not to listen to or trust official government information [6].

According to the WHO, SARS-CoV-2 is transmitted during close contact via respiratory droplets (e.g., during coughing) and by fomites [7]. To prevent the transmission of SARS-CoV-2, the WHO recommends frequent hand hygiene, use of respiratory protection, regular cleaning and disinfection of surfaces, maintenance of physical distances, and avoidance of people with fever or respiratory symptoms [2]. Based on these recommendations, most countries have addressed the outbreak according to local circumstances and have attempted to manage the accompanying economic downturn. However, the development of further strategies for greater control of the pandemic is required.

Appropriate provision of accurate information is essential if governments are to encourage citizens to follow appropriate behaviors to prevent the spread of COVID-19. However, there are regional differences in public responses to the pandemic [8]. There are also international differences in public cognitive and behavioral patterns, attitudes toward information sources, and anxiety about the COVID-19 pandemic. Information about these differences could contribute to our understanding of patterns of epidemic/pandemic-related anxiety and behavior, and would help optimize future policies for preventing the next wave of the epidemic.

## Materials and methods

We conducted a series of web-based cross-sectional surveys to determine international differences in the perceptions and behaviors of the population in the current COVID-19 risk situation. We asked a market research company, Cross Marketing Group Inc., to recruit a total of 8,000 individuals for this series of surveys, which were conducted in two stages. Participants had to be over 20 years old. As the study aim was to examine individuals’ perception and anxiety regarding COVID-19, we excluded people who had experience of COVID-19 infection as they may have obtained substantial information about COVID-19 from their doctor. In the first stage, we conducted a survey of 4000 residents of Japan. The results were independently analyzed and published [6]. These data are used as a reference in the present report. In the second stage, we conducted a survey of 2,000 individuals in the UK and 2,000 individuals in Spain.

As this was an exploratory study, no specific hypothesis was tested. Therefore, we could not calculate the minimum required sample size. However, an estimate of 500 samples from each region is considered adequate for interregional comparison studies, and multivariate analysis requires generally ten-fold of the number of variables [9]. After consideration of the size of the research budget, we chose to obtain 2000 samples from the UK and 2000 from Spain. We chose the UK because it provides a good contrast to Japan; the UK is an island, like Japan, but has experienced a high COVID-19 infection rate. Spain was chosen as an appropriate contrast to the UK, as it has a similar high infection rate but different political and geographical characteristics. The sample sizes were calculated according to the number of questionnaire items and the feasibility of rapid data gathering. In this report, we present the data from the UK and Spain combined with those from Japan.

We used questions from a previous study [2] but added some new questions about anxiety about symptom aggravation and virus transmission. In addition to items on demographic information, the questionnaire included several items assessing levels of fear and anxiety about COVID-19-related issues, the frequency of respondents' media exposure and trust in each media source, and frequency of anti-infection behaviors. Participants provided answers on a scale from 1 (none/never) to 9 (extremely/strongest) for items about symptoms understanding, preventive methods, health management and consulting health services if infected, levels of fear and anxiety, and the frequency of anti-infection behaviors. Items measuring the frequency of media exposure and degree of trust in each media source were rated on a scale from 1 (almost none/not at all) to 5 (very/greatly). We also asked the UK and Spain participants about the frequency of hand washing in their daily life. The questionnaire is shown in Additional file 1. We did not ask Japanese participants how many times a day they washed their hands (questionnaire item F(a)), because this had been measured by a previous Japanese survey (shown in Additional file 1) [10].

We analyzed the data using SPSS for Windows, ver. 24 (IBM, Armonk, NY, USA). We used the *χ*^2^-test to analyze nominal data, analysis of variance (ANOVA) with the Games–Howell test for parametric data, and the Kruskal–Wallis test with Dunn–Bonferroni correction for non-parametric data. The level of significance was set at *p* < 0.05.

The study protocol was approved by the ethics committee of Chiba University Graduate School of Medicine and the ethics committee of the International University of Health and Welfare before implementation. Participants were informed that their participation was voluntary. We did not gather any personally identifiable information about the responders. The participants were rewarded according to the regulations of Cross Marketing Group Inc.

## Results

Between March 27 and 28, 2020, a total of 4000 participants in Japan stratified by age (20s, 30s, 40s, 50s, and ≥ 60 years) and gender (400 in each group) took part in this study. Between April 17 and 21, 2020, a total of 2000 participants in the UK and 2000 participants in Spain stratified by age (20s, 30s, 40s, 50s, and ≥ 60 years) and gender (200 in each group) took part in this study. Current demographic and COVID-19 data for each country are shown in Tables 1 and 2.

**Table 1:** The participants’ demographic characteristics

	Japan		UK		Spain	
	*n*	%	*n*	%	*n*	%
Age						
20 s	791	19.9	393	19.8	396	19.8
30 s	793	19.9	398	20.0	400	20.1
40 s	800	20.1	399	20.1	399	20.0
50 s	799	20.1	399	20.1	400	20.1
≥ 60 s	798	20.0	398	20.0	400	20.1
Total	3981	100	1987	100	1995	100
Gender						
Male	1984	49.8	993	50.0	997	50.0
Female	1997	50.2	994	50.0	998	50.0
Total	3981	100	1987	100	1995	100
Educational background						
Junior high school/Secondary school	106	2.7	258	13.0	379	19.0
High school/A-level or equivalent	1223	30.7	601	30.2	242	12.1
Diploma course or vocational school	876	22.0	306	15.4	526	26.4
University degree or above	1776	44.6	822	41.4	848	42.5
Total	3981	100	1987	100	1995	100
Infected family or relatives						
Yes	17	0.4	498	25.1	779	39.0
No	5909	95.1	1183	59.5	939	47.1
Uncertain	177	4.4	306	15.4	277	13.9
Total	3981	100	1987	100	1995	100
Infected persons in your workplace						
Yes	45	1.1	342	17.2	377	18.9
No	3744	94.0	1263	63.6	1278	64.1
Uncertain	192	4.8	382	19.2	340	17.0
Total	3981	100	1987	100	1995	100

**Table 2:** Situations in each nation at the time of survey

			Japan	UK	Spain
Population (per million, 2019)			127	66.7	47.0
Date of the survey (2020)			27–28 March	17–21 April	17–21 April
No. of participants			4000	2000	2000
Valid answers			3981	1987	1995
Data at the first day of the survey:					
Daily COVID-19 tests per thousand people			0.01 (on 28 March)	0.18	0.23 (on 20 April)
Cumulative infected people with COVID-19			1,499	108,692	190,839
	Per capita (/1,000,000)		11.8	1630	4060
Total no. of fatal cases with COVID-19			49	16,879	19,478
	Per capita (/1,000,000)		0.386	253	414
Social situation		Lockdown	No	Govt. order	Govt. order
		Restricted businesses	No	Govt. order	Govt. order
		Restricted going out	Voluntary	Govt. order	Govt. order

The data were extracted from the following: Google.com http://www.google.com Worldmeters.info https://www.worldometers.info/coronavirus/country/uk/

Our World in Data https://ourworldindata.org/coronavirus-testing

We first excluded from the analysis all invalid answers, as non-serious responses may have been provided by individuals who participated in the survey just for reward. Participants who scored 1 on all items in sections C, D, E, and F (except those assessing frequency of hand washing) were excluded. As a result, 19 participants in Japan, 13 in the UK, and 5 in Spain were excluded from this analysis. Data for the remaining 7963 participants were analyzed.

Participant' demographic data are shown in Table 1. Regarding educational background, a lower percentage of participants in Japan had university degrees than those in the UK and Spain (*χ*^2^–test, Pearson *χ*^2^ = 674.390, df = 6, *p* < 0.001, adjusted residual for university degree or above for participants in Japan = 2.4). Compared with participants in the UK and Spain, a lower percentage of those in Japan had infected family or relatives (*χ*^2^–test, Pearson *χ*^2^ = 2055.999, df = 4, *p* < 0.001, adjusted residual for participants in Japan who answered “yes” =  − 38.3) or work colleagues (*χ*^2^–test, Pearson *χ*^2^ = 1139.305, df = 4, *p* < 0.001, adjusted residual for participants in Japan who answered “yes” =  − 18.6).

**Fig. 1:**
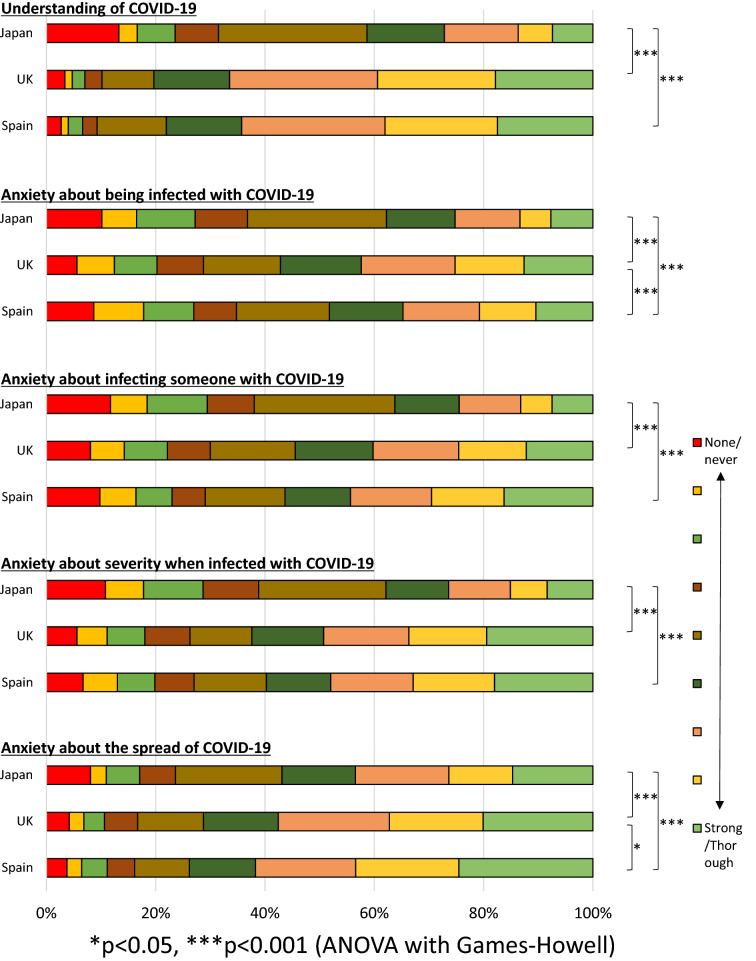
Knowledge and anxiety about COVID-19 in each country

Regarding the level of knowledge about COVID-19, there were definite and significant differences between the three countries, as shown in Fig. 1. Participants in the UK and Spain had a deeper understanding of COVID-19 than those in Japan (ANOVA with Games–Howell test), at least as reflected in their subjective responses.

There were also significant between-country differences in anxiety about COVID-19 (Fig. 1). Participants in Spain were more anxious about being infected with COVID-19 than those in Japan, but less anxious than those in the UK. Participants in Japan were less afraid of infecting others with COVID-19 than those in the UK and Spain. Regarding anxiety about the severity of the disease once infected, participants in Japan were less concerned than those in the UK and Spain. Concerning the spread of COVID-19, participants in Spain were the most anxious, followed by those in the UK and then Japan.

**Fig. 2:**
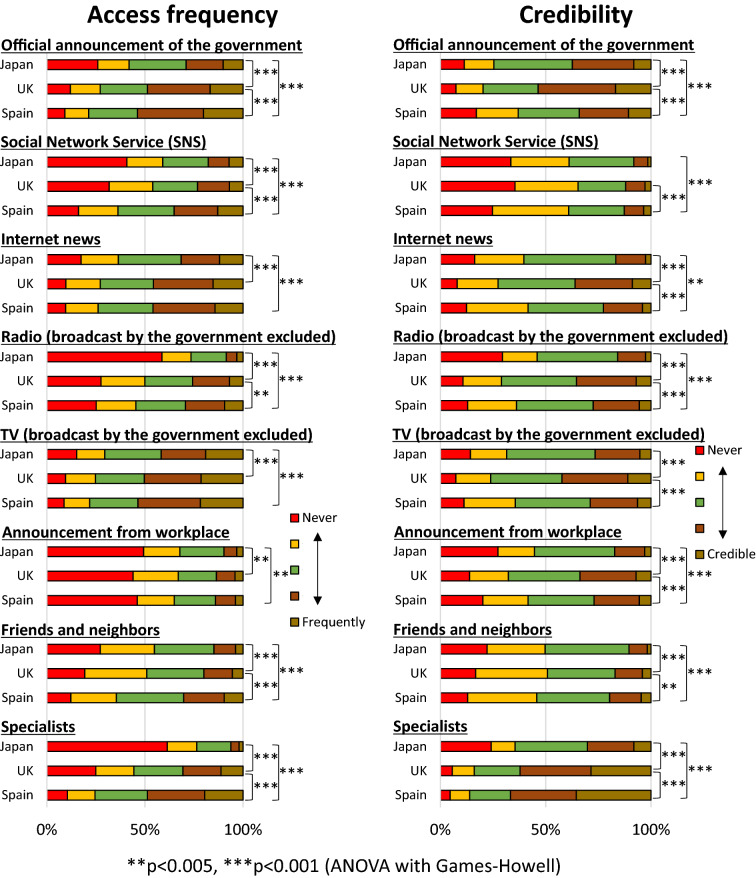
Attitude toward information sources in each country

We examined the frequency of access to and the credibility of a variety of information sources about the virus and pandemic. The survey responses revealed some country-specific characteristics (Fig. 2). Participants in Japan reported significantly less frequent access to any form of information compared with those in the UK and Spain. Remarkably, the proportion of participants in Japan who said that they had never accessed official announcements, radio, or specialists was twice as high as that of the other two countries. Participants in Spain had more frequent access than those in the UK to information sources, such as the government, a social network, radio, friends and neighbors, and specialists.

Regarding the credibility of COVID-19 information sources, participants in Japan were unlikely to trust most types of information source than those in the UK and Spain. Participants in Spain expressed less trust in government information than participants in Japan and the UK. Social networks and friends and neighbors were not deemed credible information sources in any of the three countries, but participants in Spain reported slightly more trust in these sources than those in Japan and the UK.

We compared the frequency of daily hand washing between countries. The hand washing frequency range was 4–5 times per day in all three countries. A Kruskal–Wallis test with Dunn–Bonferroni correction revealed that participants in Spain washed their hands significantly fewer times over the course of a day than participants in the UK (*p* < 0.001). However, the frequency of hand washing for participants in Spain was greater than that reported for participants in Japan in a previous survey (*p* < 0.001) [10].

**Fig. 3:**
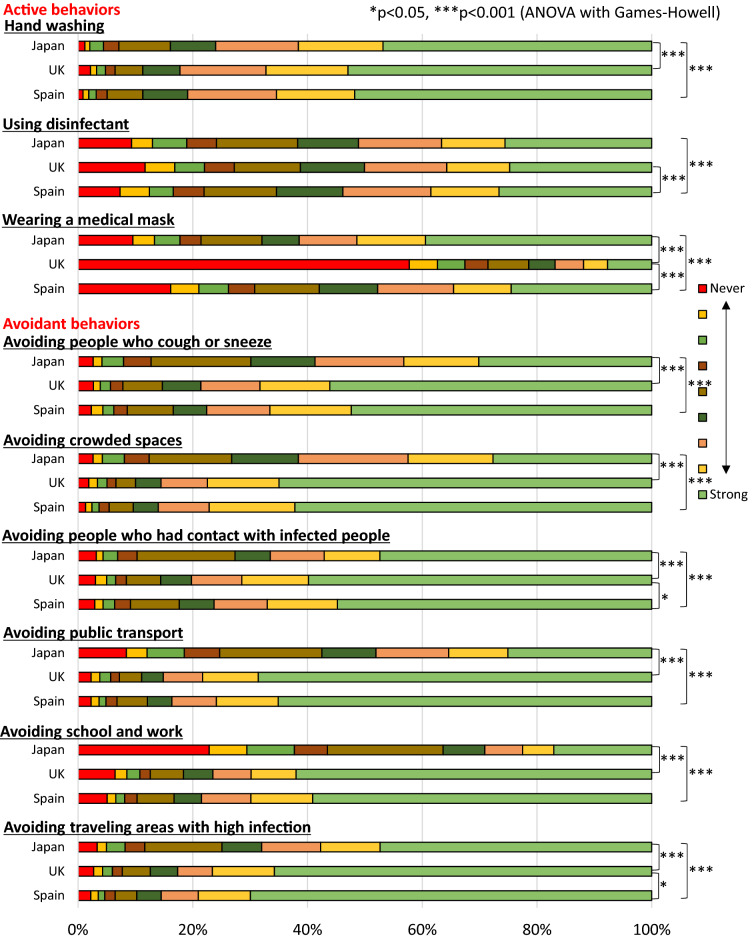
Frequency of precautionary behaviors in each country

Responses to items on precautionary behaviors are shown in Fig. 3. Among the active behaviors, patterns of hand washing and disinfectant use showed significant international differences, but these differences were complex. For example, some UK respondents washed their hands very frequently, although there were more respondents in the UK who never washed their hands compared with Spain and Japan. We also observed that 57.7% of UK participants never wore a medical mask compared with 16.1% and 9.5% of those in Spain and Japan, respectively.

We conducted an additional analysis to clarify the factors why participants in the UK avoided wearing a medical mask. A stepwise linear regression analysis was conducted in which frequency of mask wearing was the dependent variable and credibility of each information source was the independent variable. The results showed that credibility of social network services (*t* = 11.840, *p* < 0.001) and friends and neighbors (*t* = 6.445, *p* < 0.001) were positively associated with mask wearing, whereas credibility of specialists (*t* = − 3.902, *p* < 0.001) and official announcement of the government (*t* = − 2.591, *p* = 0.010) were negatively associated. In Spain, credibility of social network service (*t* = 4.462, *p* < 0.01), friends and neighbors (*t* = 3.253, *p* = 0.001), specialists (*t* = 2.667, *p* = 0.008), and TV (*t* = 2.253, *p* = 0.024) were positively associated with mask wearing frequency.

Participants in Japan were less likely to engage in avoidance behaviors than those in the UK and Spain. Notably, participants in Japan were far less likely to avoid school or work and less likely to avoid using public transport than respondents in other countries. Most participants in the UK and Spain reported that they rarely or never went to school or work, or used public transport.

As an additional analysis, we compared data on the level of knowledge, anxiety, frequency and credibility of virus information, and precautionary behaviors between participants who had acquaintances affected by the virus and those who did not. Almost every item on the questionnaire showed a significant difference between these two groups.

We reanalyzed international differences in the items presented above by examining only participants who did not have an infected acquaintance. However, this made little difference to the results.

## Discussion

We conducted an international online series of questionnaires involving a total of 8,000 individuals to investigate their knowledge, anxiety, protective behaviors, and access to information regarding COVID-19 during early spring 2020.

The data indicated that participants in the UK and Spain had a deeper understanding of COVID-19 than those in Japan. Participants in the UK and Spain also showed greater anxiety about each item than those in Japan. The frequency of access to sources of COVID-19 information was also significantly higher in the UK and Spain than in Japan.

The differences in the attitude of residents of the three countries toward COVID-19 can be explained mainly by differences in the current pandemic situation in each country. At the time of this investigation, the spread of COVID-19 in Japan was limited to several areas, but a few days after the questionnaire was administered, the number of infected people rose sharply, and the government declared a state of emergency (although in some prefectures, it was announced on April 7 [11] that residents were highly recommended to stay their homes unless it was urgent). After the end of May, the government began to gradually relax the restrictions [12]. In contrast, during the period of questionnaire administration in the UK, the spread of COVID-19 in the UK was much more severe than that in Japan. Residents were required to stay home unless there was an essential need to go out. Public business activities were prohibited, with few exceptions, until the government relaxed the restrictions on May 11, 2020 [13]. The number of deaths from COVID-19 increased linearly during the questionnaire period. In Spain, a very strict lockdown had been in place since March 14, 2020. Citizens were forbidden from leaving their homes unless necessary to obtain food or medical care, or to walk dogs. The Spanish government relaxed this regulation after a drop in the number of new infection [14], but people were required to wear face masks on public transport [15]. However, the government subsequently declared another state of emergency. To summarize, it is possible that a higher infection rate increases both anxiety and an awareness of the need for knowledge about the virus, driving the need for more precise information.

Similar international surveys have been conducted recently by other organizations. Dryhurst et al. [16] conducted an international comparison study of the perception of COVID-19 risk; they found that respondents in the UK had a higher level of risk perception than those in Japan and Spain. These results are consistent with the present findings. Gallup International also conducted a series of international surveys. The results showed that people in Japan were less likely to be anxious about catching COVID-19 than those in the UK, whereas more Japanese people reported being very or somewhat scared about COVID-19 during the period in which our questionnaire was administered [17]. Our results are generally consistent with those of other concurrent surveys.

Regarding respondents precautionary behaviors, we observed differences in the response patterns for active behaviors. Respondents in the UK and Spain more frequently washed their hands than those in Japan before the pandemic (as reported previously). To the best of our knowledge, no other reports have conducted an international comparison of three countries on hand washing frequency. It remains to be cleared why UK respondents wash their hands significantly more frequently than those in Spain.

We found notable differences in mask wearing between the countries. UK participants were far less likely to wear a mask. This difference cannot be explained by the extent of infection spread; rather, it may reflect each country’s government policy and culture [18]. The WHO did not recommend that healthy people wear a face mask unless there are clear reasons to do so [18]. The UK government did not encourage citizens to wear face masks, as there is little evidence of the effectiveness of masks for preventing infection [18, 19]. In contrast, it is well known that Japanese people are willing to wear masks partly because many individuals experience spring allergies. Approximately 40% of Japanese university students wear masks in the spring, according to a previous survey [20]. The high percentage of mask wearing in Japan before the rapid increase in symptomatic COVID-19 cases may have contributed to the reduction in the number of infectious cases, because wearing a mask can prevent splash infection by blocking saliva containing the virus (although it does not block the coronavirus itself) [21]. However, a recent report indicates that many SARS-CoV-2 transmissions occur from presymptomatic carriers [22]. Another recent study described the efficacy of medical mask wearing by asymptomatic individuals as well as lockdown at the population level [23].

We observed that participants in Japan were significantly less likely to engage in any of the avoidance behaviors examined than those in Spain and the UK. These results are similar to those of other concurrent surveys [17]. Leaving one’s home and working outside were seriously restricted in the UK and Spain at the time of our survey, whereas in Japan, only residents in specific regions were advised (not mandated) to stay home. Workplaces were not closed in many parts of Japan at the time of this study. These situational discrepancies contributed to the differences in social activities between the countries.

A survey of Italian subjects indicated that a higher level of knowledge was positively associated with the acceptance of strict mitigation measures, such as lockdown [24]. If this finding is applicable to people in other countries, we should expect people in the UK and Spain to be more supportive of national lockdown policies than those in Japan, because the present findings indicate that the former populations have higher risk perception and anxiety. In addition, UK respondents reported a high level of trust in official information, which is consistent with a Gallup International report that UK respondents were likely to believe that their government was handling the coronavirus situation well [17]. However, our results indicate that the study population in Spain tended not to trust official information.

Our Spanish respondents were least likely to trust the government, followed by those in Japan; instead, they tended to trust specialists as a source of information. Japanese respondents trusted very few sources of information about the pandemic. Cultural factors and recent political conflicts may have contributed to these differences in basic trust in officials. It was reported very recently that many individuals in England believe conspiracy theories about COVID-19, and that these theories are associated with less adherence to official guidelines for precautionary behaviors [25]. We observed that respondents who had an acquaintance who was infected with the virus were likely to trust official information about COVID-19. These results suggest that Japanese residents may be vulnerable to conspiracy theories partly because of the (relatively rare) experience of having an infected acquaintance, although there is no evidence for the spread of COVID-19 conspiracy theories in Japan.

There are several potential study limitations. The study was conducted from March to April, 2020. The survey period differed between Japan and the other countries, which may have influenced the results. The standard of COVID-19 testing also varied between countries. In addition, as a series of web-based questionnaires was used, we cannot rule out the possibility of selection bias among participants. We obtained responses only from individuals willing to complete online questionnaires. According to the Organisation for Economic Co-operation and Development, Internet services were available for 95.8 and 91.4% of citizens in the UK and Spain, respectively, in 2019 [26]. In Japan, 79.8% of citizens used Internet services in 2019, according to the Ministry of Internal Affairs and Communications [27]. However, our participants may not be representative of the Internet users in each country because only people who had registered with the monitor database of the Cross Marketing Group Inc. Additionally, participants’ residential areas may have affected the perception of COVID-19. As we did not record the residential area of participants in the UK and Spain, we could not examine the effect of area on anxiety and behavior.

## Conclusions

Despite these limitations, we believe that our findings could inform policy making to control the current pandemic situation. The COVID-19 pandemic has caused widespread public anxiety. However, moderate levels of fear of infection can encourage the public to engage in protective behaviors. Therefore, it is important that the government and other authorities provide precise unbiased information (that is not overly optimistic) about the seriousness of the current situation.

Our findings indicate several international differences in public perception and behaviors during the COVID-19 pandemic. These differences suggest the need to establish region- and country-specific solutions. For example, in Spain, providing accurate information through non-official channels, such as social network services, would be beneficial, because many people do not trust official announcements. In contrast, compensation for absence from work may encourage people in Japan to avoid work and public transport. In the UK, the low rate of mask wearing and relatively high trust in the government suggest that an official framework to encourage individuals to wear medical masks would help mitigate the spread of COVID-19.

## Supplementary Information

**Additional file 1.** This file is the whole content of the questionnaire.

## Data Availability

All data generated or analyzed during this study are included in this published article.

## Abbreviations

ANOVA: Analysis of variance
COVID-19: Coronavirus disease 2019
SARS-CoV-2: Severe acute respiratory syndrome coronavirus 2
UK: United Kingdom
WHO: World Health Organization
