# Supplementary material for: Perception of and anxiety about COVID-19 infection and risk behaviors for spreading infection: an international comparison
Source: Ann Gen Psychiatry. 2021 Feb 18;20:13. doi: 10.1186/s12991-021-00334-6 (PMC7890773; doi:10.1186/s12991-021-00334-6)
Supplement: Supplementary file 1 — Additional file 1. This file is the whole content of the questionnaire. [file 12991_2021_334_MOESM1_ESM.docx]

**Survey on** **the Relationship between Anxiety and Infection Prevention Behaviors in** **Prevalence of Infectious Disease**

(For web survey participants)

We are currently conducting a survey on relationships between anxiety and infection prevention behaviors in prevalence of infectious disease in different countries. We would like to ask you to complete the following questionnaire. The results will help us to develop epidemic control strategies in the future.

**1. Project Name:** Survey on Relationships between Anxiety and Infection Prevention Behaviors in Prevalence of Infectious Disease

**2. Purpose of the research**

The acute prevalence of the new infectious disease causes anxiety and behaviors to prevent the infection, but the level of anxiety and the frequency and intensity of the behaviors may vary from individual to individual or from country to country. The purpose of this study is to investigate the relationship between anxiety about new type coronavirus infection (COVID-19) and infection prevention behaviors in different countries. We believe that the results of this study will be useful for the development of the strategies for epidemic control in the future.

**3. Participants and method**

You will be asked to respond to questions about your anxiety about COVID-19 infection, and behaviors to prevent its transmission, age, gender and etc. If you have been diagnosed with COVID-19 infection, you are unable to take part in this study. By responding, you consent to your participation in the study. It will take 10-15 minutes to complete the questionnaire. If you feel uncomfortable, please withdraw from answering. Participation is voluntary and you will not have any disadvantage if you decide not to take part.

**4. Ethical considerations and handling of personal information**

This study was approved by the Ethics Committee of the Graduate School of Medicine, Chiba University. There are no personally identifiable questions in the survey contents. We do not know who took part, and we do not disclose the names of those who took part when publishing research results. Data will be stored properly in the laboratory, Department of Psychiatry, Graduate School of Medicine, Chiba University.

**Principal Investigator**:

Masaomi Iyo, MD, PhD., Professor and Chairman of Department of Psychiatry, Graduate School of Medicine, Chiba University

**Contact:**

Akihiro Shiina, MD, PhD., a Research Associate Professor of Center for Forensic Mental Health, Chiba University

**Screening Question**

(1) Have you ever been diagnosed with COVID-19 infection?

Yes No

*** If "No", please answer the following questions.**

1. **Demographic characteristics.**

Please select your age group.

1) Age: 20s 30s 40s 50s 60s 70s 80 years and over

2) Gender : 1. Male 2. Female

3) Educational background: 1. Junior high school/Secondary School 2. High school/A-levels or equivalent 3. Diploma course or vocational school. 4. University degree or above

**B. Confirmation of COVID-19 infection**

Please select one item for each question.

1) Has anyone of your family, friends, neighborhood, etc. been infected with COVID-19?

1. Yes 2. No 3. unknown

2) Has anyone been infected with COVID-19 at your facility, such as workplace or school?

1. Yes 2. No 3. unknown

**C. The level of knowledge and anxiety about COVID-19 infection**

Please select one item for each question. Please note that "1" means "none", 9 means "thorough” /”very strong", with "5" being in the middle.

1) Knowledge of COVID-19 (infection prevention methods, symptoms, countermeasures when infected, and consulting service)

1 None ... 2 ... 3 ... 4 ... 5 (middle) ... 6 ... 7 ... 8 ... 9 Thorough knowledge

2) The level of your anxiety that you will be infected with COVID-19

1 None ... 2 ... 3 ... 4 ... 5 (middle) ... 6 ... 7 ... 8 ... 9 Very strong

3) The level of your anxiety that you may infect someone with COVID-19

1 None ... 2 ... 3 ... 4 ... 5 (middle) ... 6 ... 7 ... 8 ... 9 Very strong

4) The level of your anxiety that it will be very severe if you become infected with COVID-19

1 None ... 2 ... 3 ... 4 ... 5 (middle) ... 6 ... 7 ... 8 ... 9 Very strong

5) The level of your anxiety about the spread of COVID-19 infection in your country

1 None ... 2 ... 3 ... 4 ... 5 (middle) ... 6 ... 7 ... 8 ... 9 Very strong

**D. Questions about** **the fear of illness**

Please indicate the level of your fear of each illness.

1) New type of coronavirus infection (COVID-19)

1 Very little 2 Little 3 Moderate 4 Strong 5 Very strong

2) Diabetes

1 Very little 2 Little 3 Moderate 4 Strong 5 Very strong

3) HIV infection

1 Very little 2 Little 3 Moderate 4 Strong 5 Very strong

4) Serious injury

1 Very little 2 Little 3 Moderate 4 Strong 5 Very strong

5) heart disease

1 Very little 2 Little 3 Moderate 4 Strong 5 Very strong

6) Cancer

1 Very little 2 Little 3 Moderate 4 Strong 5 Very strong

7) Avian influenza infection

1 Very little 2 Little 3 Moderate 4 Strong 5 Very strong

8) Seasonal influenza infection

1 Very little 2 Little 3 Moderate 4 Strong 5 Very strong

**E. Source of information on the prevalence of COVID-19 infection**

The sources of information on the prevalence of COVID-19 infection are listed below. Over the past two weeks, please select one item regarding "How often have you used the source? " and "How reliable do you think the information is?"

1) Public institutions such as national and/or local governments (including those via the mass media)

Frequency: 1 (almost none) 2 Occasionally 3 Moderately 4 Frequently 5 Very

Reliability: 1 (not at all) 2 To some extent 3 Moderately reliable 4 reliable 5 Greatly reliable

2) Social network services (SNS)

Frequency: 1 (almost none) 2 Occasionally 3 Moderately 4 Frequently 5 Very

Reliability: 1 (not at all) 2 To some extent 3 Moderately reliable 4 reliable 5 Greatly reliable

3) Online news (excluding reports from public institutions such as the national and/or local government)

Frequency: 1 (almost none) 2 Occasionally 3 Moderately 4 Frequently 5 Very

Reliability: 1 (not at all) 2 To some extent 3 Moderately reliable 4 reliable 5 Greatly reliable

4) Radio (excluding reports from public institutions such as the national and/or local government)

Frequency: 1 (almost none) 2 Occasionally 3 Moderately 4 Frequently 5 Very

Reliability: 1 (not at all) 2 To some extent 3 Moderately reliable 4 reliable 5 Greatly reliable

5) TV (excluding reports from public institutions such as the national and/or local government)

Frequency: 1 (almost none) 2 Occasionally 3 Moderately 4 Frequently 5 Very

Reliability: 1 (not at all) 2 To some extent 3 Moderately reliable 4 reliable 5 Greatly reliable

6) Information leaflet given by workplaces and schools

Frequency: 1 (almost none) 2 Occasionally 3 Moderately 4 Frequently 5 Very

Reliability: 1 (not at all) 2 To some extent 3 Moderately reliable 4 reliable 5 Greatly reliable

7) What family, friends, acquaintances, etc. tells to you

Frequency: 1 (almost none) 2 Occasionally 3 Moderately 4 Frequently 5 Very

Reliability: 1 (not at all) 2 To some extent 3 Moderately reliable 4 reliable 5 Greatly reliable

8) What specialists (doctors, nurses, etc.) tells to you

Frequency: 1 (almost none) 2 Occasionally 3 Moderately 4 Frequently 5 Very

Reliability: 1 (not at all) 2 To some extent 3 Moderately reliable 4 reliable 5 Greatly reliable

**F. Behaviors you take to prevent COVID-19 infection**

First, please think about the way you wash your hands in general.

1. Number of hand washing:

How many times a day do you usually wash hands?

1. None 2. Once 3. Twice-three times 4. Four-five times 5. Six-seven times 6. Eight-nine times 7. Ten-twelve times 8. Thirteen-fourteen times 9. Fifteen times or more 10. More than 0, but number unknown

Given the current status of the epidemic, which of the following precautionary actions have you taken over the past 2 weeks? Please select the applicable number.

1. **Positive action:**

In the order of "1" to "9", the size increases from "Never" to "Many times". "5" is in the middle.

1) Hand washing

1 Not at all ... 2 ... 3 ... 4 ... 5 (middle) ... 6 ... 7 ... 8 ... 9 Many times

2) Using disinfectant (such as alcohol disinfection)

1 Not at all ... 2 ... 3 ... 4 ... 5 (middle) ... 6 ... 7 ... 8 ... 9 Many times

3) Wearing a medical mask

1 Not at all ... 2 ... 3 ... 4 ... 5 (middle) ... 6 ... 7 ... 8 ... 9 Many times

1. **Avoidance behaviors:**

From "1" to "9", the size increases from "Never avoid" to "Avoid greatly". "5" is in the middle.

1) Avoiding people who cough or sneeze

1 Never... 2 ... 3 ... 4 ... 5 (middle) ... 6 ... 7 ... 8 ... 9 Avoid greatly

2) Avoiding places where a large group of people come together

1 Never... 2 ... 3 ... 4 ... 5 (middle) ... 6 ... 7 ... 8 ... 9 Avoid greatly

3) Avoiding people who have come in contact with infected people

1 Never... 2 ... 3 ... 4 ... 5 (middle) ... 6 ... 7 ... 8 ... 9 Avoid greatly

4) Avoiding public transport

1 Never... 2 ... 3 ... 4 ... 5 (middle) ... 6 ... 7 ... 8 ... 9 Avoid greatly

5) Avoiding school and work

1 Never... 2 ... 3 ... 4 ... 5 (middle) ... 6 ... 7 ... 8 ... 9 Avoid greatly

6) Avoiding traveling to areas with high infection

1 Never... 2 ... 3 ... 4 ... 5 (middle) ... 6 ... 7 ... 8 ... 9 Avoid greatly

The result of Dimsdrive’s survey conducted in 2015 in Japan

| Daily hand washing | Percentage |
| --- | --- |
| Once | 2.6 |
| Twice or three times | 18.1 |
| 4–5 | 25.0 |
| 6–7 | 13.9 |
| 8–9 | 6.8 |
| 10–12 | 12.7 |
| 13–14 | 1.0 |
| 15 or more | 7.6 |
| Unknown | 11.3 |
| Never | 1.0 |

2,775 male and 1,756 female participants answered the questionnaire via the internet.
